# Supplementary material for: Reduced Expression of Annexin A6 Induces Metabolic Reprogramming That Favors Rapid Fatty Acid Oxidation in Triple-Negative Breast Cancer Cells
Source: Cancers (Basel). 2022 Feb 22;14(5):1108. doi: 10.3390/cancers14051108 (PMC8909273; doi:10.3390/cancers14051108)
Supplement: Supplementary file 1 [file cancers-14-01108-s001.zip › cancers-1590119-supplementary.pdf]

cancers

Supplementary Material: Reduced Expression of Annexin A6 Induces Metabolic Reprogramming That Favors Rapid Fatty Acid Oxidation in Triple-Negative Breast Cancer Cells

Stephen D. Williams and Amos M. Sakwe

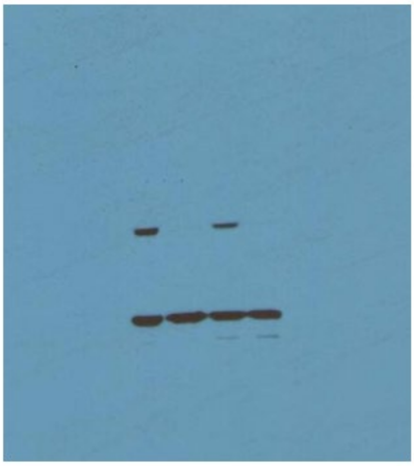

Lane 1: BT-NSC

Lane 2: BT-A6sh5

Lane 3: MDA-NSC

Lane 4: MDA-A6sh5

Figure S1. The original western blots for figure 1A.

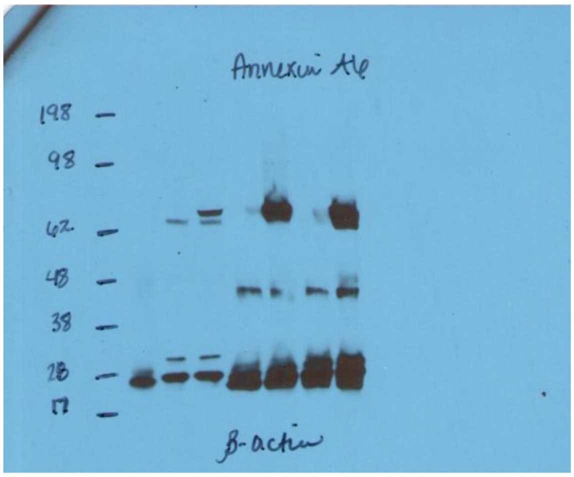

Lane 2: MDA-468 EV

Lane 3: MDA-Flag-A6

Figure S2. The original western blots for figure 2A.

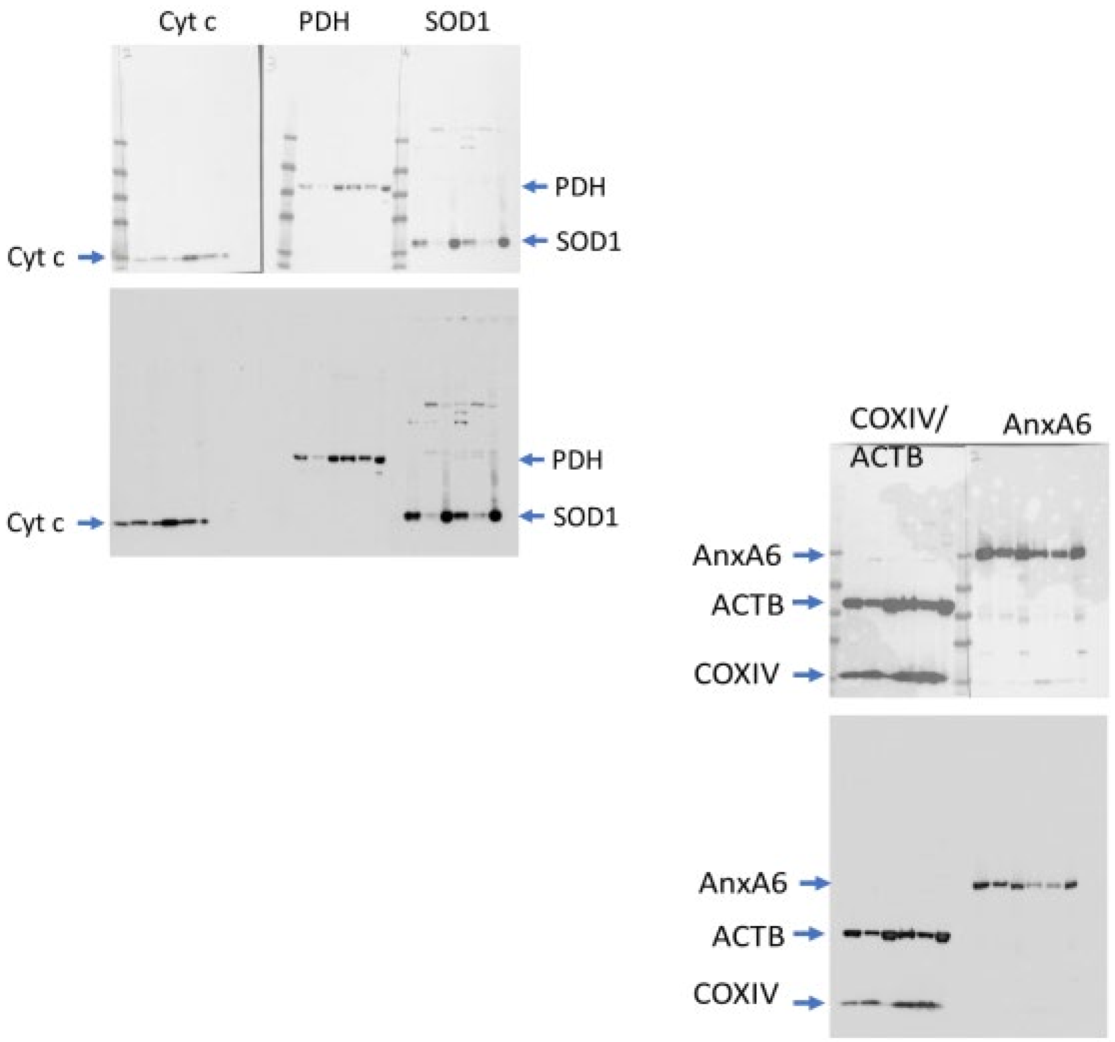

Figure S3. The original western blots for figure 3A.

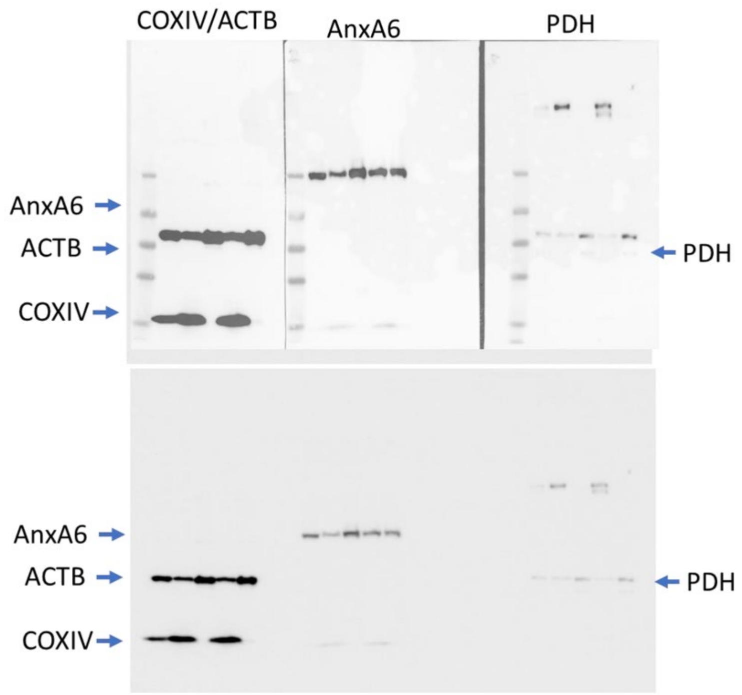

Figure S4. The original western blots for figure 3C.

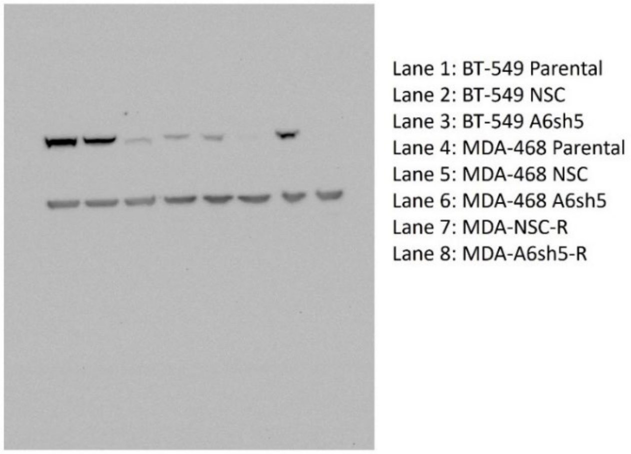

Figure S5. The original western blots for figure 5A.
